# Fears and Beliefs in Rheumatoid Arthritis and Spondyloarthritis: A Qualitative Study

**DOI:** 10.1371/journal.pone.0114350

**Published:** 2014-12-04

**Authors:** Francis Berenbaum, Pierre Chauvin, Christophe Hudry, Florence Mathoret-Philibert, Maud Poussiere, Thibault De Chalus, Caroline Dreuillet, Françoise Russo-Marie, Jean-Michel Joubert, Alain Saraux

**Affiliations:** 1 Sorbonne Universités, UPMC Univ Paris 6, AP-HP, Hôpital Saint-Antoine, Rheumatology Department, Paris, France; 2 INSERM, Sorbonne Universités, UPMC Univ Paris 6, UMR_S 1136, Pierre Louis Institute of Epidemiology and Public Health, Department of Social Epidemiology, Paris, France; 3 AP-HP, Hôpital Pitié-Salpétrière, Rheumatology Department, Paris, France; 4 AP-HP, Hôpital Cochin, Clinical Psychology Department, Paris, France; 5 Independent Researcher, Paris, France; 6 UCB Laboratoires, Colombes, France; 7 Arthritis Fondation Courtin, Neuilly-sur-Seine, France; 8 CHU La Cavale Blanche, Rheumatology Department, Brest, France; University of Birmingham, United Kingdom

## Abstract

**Objectives:**

To explore beliefs and apprehensions about disease and its treatment in patients with rheumatoid arthritis and spondyloarthritis.

**Methods:**

25 patients with rheumatoid arthritis and 25 with spondyloarthritis participated in semi-structured interviews about their disease and its treatment. The interviews were performed by trained interviewers in participants' homes. The interviews were recorded and the main themes identified by content analysis.

**Results:**

Patients differentiated between the underlying cause of the disease, which was most frequently identified as a hereditary or individual predisposition. In patients with rheumatoid arthritis, the most frequently cited triggering factor for disease onset was a psychological factor or life-event, whereas patients with spondyloarthritis tended to focus more on an intrinsic vulnerability to disease. Stress and overexertion were considered important triggering factors for exacerbations, and relaxation techniques were frequently cited strategies to manage exacerbations. The unpredictability of the disease course was a common source of anxiety. Beliefs about the disease and apprehensions about the future tended to evolve over the course of the disease, as did treatment expectations.

**Conclusions:**

Patients with rheumatoid arthritis and spondyloarthritis hold a core set of beliefs and apprehensions that reflect their level of information about their disease and are not necessarily appropriate. The physician can initiate discussion of these beliefs in order to dispel misconceptions, align treatment expectations, provide reassurance to the patient and readjust disease management. Such a dialogue would help improve standards of care in these chronic and incapacitating diseases.

## Introduction

Rheumatoid arthritis (RA) and spondyloarthritis (SpA) are the two most frequent chronic inflammatory joint diseases, with a prevalence of around 0.3% in the French adult population [1], [2]. Whereas RA affects mainly the peripheral joints, SpA usually affects the spine, as well as sacroiliac and hip joints. Both diseases are characterised by a fluctuating course, in which exacerbations (flares) are separated by periods of improvement of variable length. Recovery from exacerbations is rarely complete, resulting in progressive irreversible structural damage to the affected joints [3], [4].

The chronicity of these inflammatory joint diseases, the variability and unpredictability of the disease course and the disabling nature of the symptoms means that these diseases have a significant negative impact on quality of life, functioning, autonomy and psychological well-being. A number of meta-syntheses of qualitative studies in RA have been published [5]–[9]. These have indicated that patient's knowledge about RA when they are diagnosed is limited and that misperceptions of causes are common [6], [7]. Daker-White and colleagues [9] have highlighted the important impact of RA on patients' self-image with fears of inadequate role fulfilment and of dependency on others, whereas Campbell et al have emphasised difficulty with coping with the unpredictability of pain and fatigue [6]. A recent systematic literature review of the subjective impact of RA and SpA on patient well-being identified a number of studies in patients with RA but relatively few in patients with SpA [10]. The majority of studies explored factors related to the physical impact of disease, notably pain, functional performance and fatigue. Others have addressed its psychological impact, including psychological distress and coping strategies. In contrast, very few studies have investigated the fears and beliefs of patients with respect to their disease, with only a few such studies being identified in patients with RA [11]–[16] and none in patients with SpA.

It is important to understand patients' attitudes and beliefs towards their disease in order to improve patient-physician dialogue, to raise the quality of care offered, to provide a more interactive and dynamic approach to therapy, and to optimise treatment adherence. For these reasons, we have undertaken the present qualitative study in order to explore patients' beliefs and apprehensions concerning their disease and its treatment. Such information may help to understand perceptions of autoimmune rheumatic diseases and their repercussions better.

## Methods

This was an exploratory qualitative study performed in France between April and July 2012. The study was carried out from the perspective of rheumatologists interested in developing patient-reported outcome measures for rheumatological disorders that may be used to improve standards of patient care. The findings are reported as a qualitative analysis of content of semi-structured interviews.

The study was conducted in two phases. The first phase, consisting of face-to-face interviews with fourteen patients, was purely exploratory. The interviewer asked open-ended questions on how patients felt and what they understood about their disease and its treatment. The rationale for this phase was that information on what aspects of the disease were most important or worrisome to the patient was limited and, for this reason, there were no *a priori* hypotheses to test. The objective of the second phase, involving 36 additional patients, was to explore in more detail the themes identified in the first phase. To this end, the interviewer, in addition to the general open-ended questions from the first phase, also asked a number of semi-directed questions to collect more details about specific beliefs and apprehensions. Around three-quarters of the total interview time was devoted to the open-ended questions. Examples of questions asked in the first and second phase of the survey are provided in (S1 Table).

### Participants

Participants in the survey were identified by hospital- or community- based rheumatologists. Each could recruit up to four patients, two with a diagnosis of RA and two with a diagnosis of SpA. Twenty rheumatologists recruited 25 patients with RA and 25 with SpA to participate in the study. These patients were to have a confirmed diagnosis of RA (2010 ACR/EULAR criteria) [17] or SpA (2011 ASAS criteria) [18] made at least six months previously, and to be at least eighteen years of age. For each disease group, it was attempted to ensure the diversity and representativeness of the sample with regard to age, gender, region of France, disease duration, symptom severity and socioeconomic status. Patients were invited to participate in the survey over the telephone, and an appointment made for the interview.

### Data collection

Following a training session about the study, each interviewer received a study guide which specified the goals of the study and the different questions to be asked. The study guide contained general recommendations about conducting the interview and specific questions on RA and SpA identified by the Steering Committee prior to the study. After the first phase of the study (fourteen interviews with open questions only), the study guide was updated based on the findings to include suggestions for semi-directed questions to be asked during the second phase of the study. Recommendations were provided to adapt the style of interview to the patient's cultural and linguistic perspective, for example using the term spontaneously used by the patient to describe their illness.

Participating patients were interviewed face-to-face at home by a trained interviewer. One-to-one interviews, which were recorded, lasted approximately 75 minutes for the first phase and approximately 125 minutes for the second phase. For each patient, demographic and clinical variables were documented by the rheumatologist from patient records. Disease activity was determined by the rheumatologist with the 28-item Disease Activity Score (DAS28) for RA and with the Bath Ankylosing Spondylitis Disease Activity Index (BASDAI) for SpA [19], [20]. The most recent evaluation available in the patient records was taken.

### Data analysis

Interviews were transcribed *verbatim*, and data were extracted inductively from the interview transcripts. Concepts were grouped by theme by the data analyst. Since this was a qualitative study, no formal coding system was used. A thematic analysis was performed on the patients' responses on a question-by-question or paragraph-by-paragraph basis, according to the amount of material [21]. Wherever possible, verbatim quotes are provided to illustrate the different ideas put forward by the patient. No feedback on individual interviews was provided to the interviewees.

## Results

### Patients

Overall, fifty patients, 25 with RA and 25 with SpA, accepted to participate in the survey. The demographic and disease features of these patients are presented in Table 1. Three-quarters of the patients with RA and half of those with SpA were women. With regard to age and disease activity in the RA patient subgroup, over half the patients had a long disease duration (>10 years) and all patients interviewed were over 35 years of age. Patients with SpA were evenly distributed across age groups and disease duration classes, although forty percent of patients interviewed had low disease activity (BASDAI score <30).

**Table 1:** Patient characteristics.

		RA		SpA
		(N = 25)		(N = 25)
**Gender**	Women	19 (76.0%)	Women	13 (52.0%)
	Men	6 (24.0%)	Men	12 (48.0%)
**Age**	≤35 years	None	≤35 years	7 (28.0%)
	36 to 45 years	9 (36.0%)	36 to 45 years	7 (28.0%)
	46 to 55 years	7 (28.0%)	46 to 55 years	6 (24.0%)
	>55 years	9 (36.0%)	>55 years	5 (20.0%)
**Disease duration**	<2 years	3 (12.0%)	<2 years	6 (24.0%)
	2 to 5 years	6 (24.0%)	2 to 5 years	6 (24.0%)
	6 to 10 years	3 (12.0%)	6 to 10 years	6 (24.0%)
	>10 years	13 (52.0%)	>10 years	7 (28.0%)
**Disease activity**	DAS28 <2.6	4 (16.0%)	BASDAI <30	10 (40.0%)
	DAS28 2.6–3.2	5 (20.0%)	BASDAI 30–45	6 (24.0%)
	DAS28 3.2–5.1	11 (44.0%)	BASDAI 50–60	6 (24.0%)
	DAS28 >5.1	4 (16.0%)	BASDAI >60	2 (8.0%)
	Not available	1 (4.0%)	Not available	1 (4.0%)
**Past or current DMARD treatment**		25 (100%)		21 (84.0%)
**Currently receiving biological agents**		14 (56.0%)		14 (56.0%)

### Interviews

All scheduled interviews were completed as planned. During the first phase of the interviews, where only open questions were asked, the issues brought up by the patients could be classified into five major themes, namely disease aetiology, exacerbations, course of the disease, impact of disease and treatments. During the second phase, when some of the questions were semi-directive, no further major themes or concepts were identified, suggesting that concept saturation had been reached. A comprehensive listing of the different themes identified is presented in Table 2.

**Table 2:** Beliefs, fears and attitudes identified in the study.

Beliefs related to the underlying cause of disease, the appearance of first symptoms, exacerbations and disease course		
***Factors responsible for outbreak of disease***	**RA**	**SpA**
Genetic factors/genetic predisposition/HLA-B27 gene	Yes	Yes
Heredity (other family members with same disease)	Yes	Yes
Familial (other family members with joint or back problems)	Yes	Yes
Inherent susceptibility (like cancer)	Yes	Yes
Autoimmune disease (*eg* Crohn's disease in a family member)	No	Yes
Something in the intestines	Yes	Yes
***Factors responsible for outbreak of disease***	**RA**	**SpA**
Psychological factors (distressing life event, stress, hyperactivity…)	Yes	Yes
Physical or tiring professional activities	Yes	Yes
Lifestyle (alcohol, smoking, diet…)	Yes	Yes
Infection/Poorly-treated infection	Yes	Yes
Environmental factor (*eg* pollution)	Yes	No
Vaccination (hepatitis B or other)	Yes	Yes
Too intense physical activity over a long period	Yes	Yes
Chronic calcium deficiency/calcification problem	Yes	No
Reiter's disease	No	Yes
***Factors triggering exacerbations***	**RA**	**SpA**
Psychological factors (stress, distressing events, low mood)	Yes	Yes
Overwork, effort, intense physical activity	Yes	Yes
Damp weather, changes in the weather	Yes	Yes
Fatigue	Yes	Yes
Diet	Yes	Yes
Poor posture	Yes	Yes
Seasonal pollen allergy	No	Yes
Alcoholic beverages	Yes	No
Treatment failure	Yes	No
***Behaviours that helped avoid or attenuate exacerbations***	**RA**	**SpA**
Oriental disciplines (yoga, chi gong, tai chi, akido, jujitsu, etc)	Yes	Yes
Sports activities or exercises (swimming, tennis, running, walking)	Yes	Yes
Paramedical care (physiotherapy, osteopathy, taking the waters)	Yes	Yes
Alternative medicine (acupuncture, homeopathy, aromatherapy)	Yes	Yes
Diet, reducing food intake	Yes	Yes
Rest	Yes	Yes
Holidays or stress-free environment	Yes	Yes
Stretching	No	Yes
Working	Yes	No
Sea air	Yes	Yes
Hot showers	Yes	Yes
Fasciatherapy	No	Yes
Hypnotism, faith healing	Yes	No
Leeches, bleeding	Yes	No
Shea balm	Yes	No
Moving to a better adapted climate	Yes	No
***Fears about the future course of the disease***	**RA**	**SpA**
Fear of paralysis/disability	Yes	Yes
Fear of dependence/loss of autonomy	Yes	Yes
Fears related to the unpredictability of the disease course	Yes	Yes
Fear of recurrent intense pain or exacerbations	Yes	Yes
Fear of physical deformation	Yes	Yes
Fear of rapid disease progression or frequent exacerbations	Yes	Yes
Fear of disease spread to other joints	Yes	No
Fear of disease spread to other organs (*eg* heart, lung, intestines)	Yes	Yes
Fear of joint fusion	No	Yes
Fear of uveitis or vision loss	No	Yes
Fear of reduced life expectancy	Yes	Yes
Fear of progression to multiple sclerosis	No	Yes
**Fears related to the impact of disease**		
	**RA**	**SpA**
Dependence/loss of autonomy	Yes	Yes
Loss of job, loss of financial security, loss of professional prospects	Yes	Yes
No longer being able to look after one's children or grandchildren	Yes	Yes
Becoming a burden for one's relatives	Yes	Yes
No longer being able to manage activities of daily living	Yes	Yes
Stigma/Being considered disabled/Becoming an object of pity	Yes	Yes
Being abandoned by partner/Having no-one to turn to	Yes	Yes
Not being able to carry through projects, plans and ambitions	Yes	Yes
Impact on social relations	Yes	Yes
Repercussions on pregnancy	Yes	Yes
Needing to move house or modify living environment	Yes	Yes
Not being able to make new close sentimental relationships	Yes	Yes
**Beliefs and attitudes about treatment.**		
***Beliefs and attitudes about side-effects of treatments***	**RA**	**SpA**
Treatments not safe in the long-term (there must be a risk)	Yes	Yes
Worries about specific side-effects	Yes	Yes
Risk of cancer (particularly skin cancer)	Yes	Yes
Lack of long-term experience with biological therapies	Yes	Yes
Shortened life expectancy	Yes	Yes
Risk of sterility	No	Yes
Risk to the foetus	Yes	Yes
***Beliefs and attitudes about efficacy of treatments***	**RA**	**SpA**
Loss of efficacy over time	Yes	Yes
Need to change treatment to something less effective	Yes	Yes
Need to stop treatment due to side-effects	Yes	Yes
Not being able to find an effective treatment	No	Yes
Being on a ‘last-chance' treatment	Yes	Yes
Belief that treatment will act immediately	No	Yes
Fear of medication being withdrawn	Yes	Yes
Not being able to pay for treatment	Yes	No

### Disease aetiology

Three themes related to disease aetiology were identified during the interview, namely beliefs about the origin of the disease, beliefs about exacerbations and apprehensions about disease progression. Concerning the underlying cause of the disease, patients frequently cited a possible hereditary factor or a familial or individual predisposition (Table 2). Patients with SpA were particularly aware of the potential role of genetic factors such as HLA-B27, whereas patients with RA were more likely to mention an external cause for their disease, such as diet or physical activity. One of the consequences of this widespread belief about hereditary was the fear of transmitting, or having already transmitted, their disease to their children, which was evoked by twenty patients with RA and twenty with SpA. Moreover, the patients generally thought that there was some lifestyle triggering factor that led to the appearance of disease if there was some hereditary or familial predisposition. Particularly in patients with RA, psychological factors, such as stress, overwork, anxiety or a distressing life event, for example bereavement or divorce, were frequently cited (Table 2). However, external factors were also cited such as diet, smoking or alcohol, vaccination or intense physical activity.

*“This is really upsetting to me as I think that perhaps my daughters will get this disease because of me and I will have given it to them.”*

Female patient with spondyloarthritis

*“I'd like to believe my doctor but I can't help thinking that losing my grandpa, doing a lot of sport and getting vaccinated all added up and that made my immune system not work properly any longer”*

Female patient with rheumatoid arthritis

*“I don't know why I have got this. I have this gene, HLA-B27, which facilitates inflammation, which favours my disease and that's it. But why it's me that gets the disease, I don't know. Why it developed like it did, I have no idea.”*

Female patient with spondyloarthritis

### Exacerbations

The triggering factors tended to be the same as those believed to have caused the disease, namely psychological factors such as stress and overwork. The belief that these were responsible for exacerbations was held more strongly than the belief that they caused the disease, since patients claimed to have direct experience that exacerbations tended to follow closely on periods of stress or fatigue. Among the other factors that were cited as triggering exacerbations were damp or changeable weather, unhealthy diet and poor posture. The protective factors cited tended to be the mirror-image of the triggering factors, namely relaxation and calm. There was a widely-held belief that anything that was good for the mind would be good for their somatic symptoms. Others, particularly patients with SpA, considered that exercise was useful to stop their joints ‘seizing up’ and took part in sports activities such as swimming, or performed stretching exercises. In addition to physiotherapy, recourse to alternative medicines was frequent. Dieting and changes to diet were also frequently cited. In most cases, these practices were started at the patients' own initiative and without any discuss*ion with their physician as to their utility.*

*“If something very stressful happens one day, then I may have an exacerbation the next day”*

*“So I stopped drinking milk because I don't think that it is good for me. I have the impression that it causes my exacerbations…. My rheumatologist doesn't agree, but I'm sure.”*

*“I do Qigong, it's very gentle, it helps me relax to concentrate and to move my whole body”*

*“I think that we should do something ourselves and not wait. (…) I think that we have to work with the medicine and do something to take charge of ourselves”*

Female patients with rheumatoid arthritis

### Course of the disease

Beliefs and expectations about the course of the disease varied according to the stage of the disease and familiarity with other people with the disease. At the time of the first symptoms or of diagnosis, patients usually imagined that they would become as handicapped as other people they knew with the disease, or if they did not have experience of the disease, become paralysed. Initially, patients frequently focussed on long-term outcomes and underestimated the everyday burden of the disease in terms of pain and fatigue. With time, these fears seemed to evolve as patients became aware of the inherent variability of the disease in terms of clinical presentation, symptom severity and course. During periods of remission or if they considered their treatments to be effective, the fear of future disability would temporarily recede. However, when an exacerbation occurred, or if the treatment stopped working, initial fears were often reactivated. The variability in the course and presentation of the disease was in itself a major source of uncertainty and worry about the future.

*“I thought that I was going to end up like my aunt, with deformed hands. But it's not like that at all.”*
*“[RA] is a chronic disease and it's going to progress as time goes by, but you don't know how. It is the ‘don't know’ that is so worrying, and the ‘when’.”*

Female patients with rheumatoid arthritis

*“I didn't think that I would suffer so much, that it would be so restricting for going to work.”*

Female patient with psoriatic arthritis

*“I don't want to be paralysed – it's what I am most frightened of. In my mind, joint fusion means being paralysed.”*
*“I know that even if I take all the medication that I possibly can, I will never get better”*

Male patients with spondylarthritis

### Impact of disease

**Figure 1:**
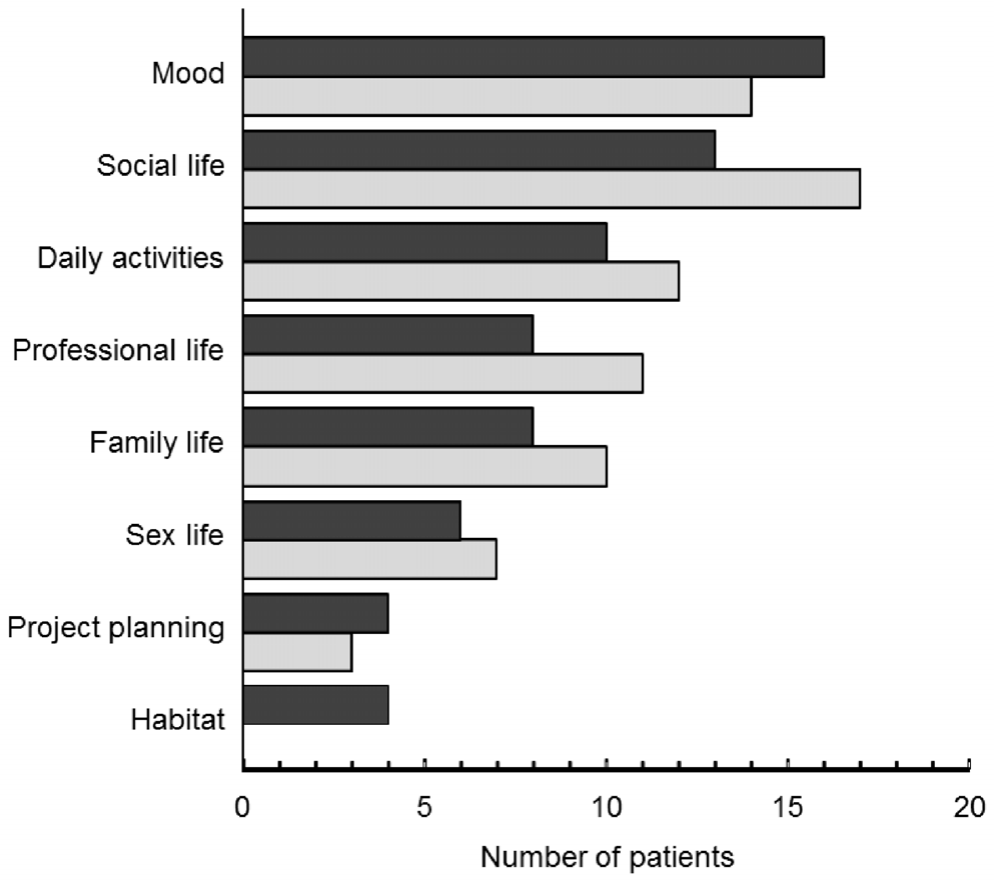
Current repercussions of disease by theme. Filled bars: patients with rheumatoid arthritis; open columns: patients with spondyloarthritis.

Patients reported both current repercussions of their disease as well as fears about the impact that the disease would have on their future lives. Current repercussions are grouped by theme in Fig. 1. Two particular repercussions were evoked by the majority of patients, namely impact on mood and impact on social life. With respect to mood, many patients felt depressed and fed up with the pain and fatigue, but also angry and irritated, particularly by the unpredictable onset of pain. This was accompanied by lowered self-esteem and a feeling of not being able to achieve what they wanted to do or what they could do before. Most patients also reported repercussions on their social life and leisure activities which would either cause them to adapt their activities (timing, duration or intensity) or give them up altogether. This latter situation was frequently a source of depression.

With respect to repercussions on activities, these covered all aspects of daily living from getting up and dressing to cooking, opening doors, driving or climbing stairs. For these everyday tasks, they often needed the help of family or friends or needed their living space to be adapted. Many commented that their entourage did not fully understand the extent of their suffering or disability, but their wish to remain independent meant that they did not always communicate their needs explicitly to their entourage.

Finally, most patients expressed fears about the future related to the impact of their disease on their life in general (Table 2). In the majority of cases, these fears related to the loss of autonomy and becoming dependent on other people. Another major theme related to social isolation, with worries about no longer being able to fulfil their social or family roles, such as looking after their children or grandchildren. Patients with RA were particularly concerned about future spread of disease to other joints, whereas those with SpA were concerned about joint fusion.

*“The state of my health is making me lose my role. On top of the physical pain, there is a psychological part.”*

*“With my friends and entourage sometimes I withdraw into my shell if I'm in pain.”*

*“Losing my independence and becoming a vegetable frighten me more than dying…”*

*“I'm ill and I know I'm going to lose my job. And that doesn't help and I worry about it. (…)Working is important for my well-being.”*

Female patients with spondylarthritis

### Beliefs and attitudes about treatment

Initially, patients thought that a treatment would stop them being in pain and even return them to the state of health that they had enjoyed before falling ill. However, following further discussion with their physician they understood that treatment would ‘just’ slow down the progression of the disease and help them be in less pain. Many apprehensions appeared about being on a ‘strong’ treatment for a long period. Many patients believed that there must be a cost in terms of side-effects for taking a medication that was very effective. This fear was reinforced by awareness that certain treatments were relatively recent and that there was limited long term experience with them. These beliefs were reinforced by having to go to the clinic regularly for monitoring of, for example, for liver enzymes or white cell counts. Such beliefs gave rise to fears that they may have to stop treatment one day due to side-effects and that they would be back in pain again.

*“No, I could never stop [my treatment]. Because I know the hell that I went through before, when every day was unbearable. Living with such pain, I couldn't do that again.”*

*“With long-term therapy, you could become tolerant. That's why I try and put off changing treatment and try to space out my injections.”*

*“It seems to me that any medication that you take for one thing is bound to upset something else.”*

Female patients with rheumatoid arthritis

## Discussion

This qualitative study has identified a series of beliefs and apprehensions commonly held by patients with RA and SpA. A striking finding was the importance given by patients to psychological factors in the development, progression and manifestations of their disease, particularly for patients with RA. In addition, a large number of patients cited psychological strategies such as relaxation techniques for avoiding or abrogating exacerbations.

**Figure 2:**
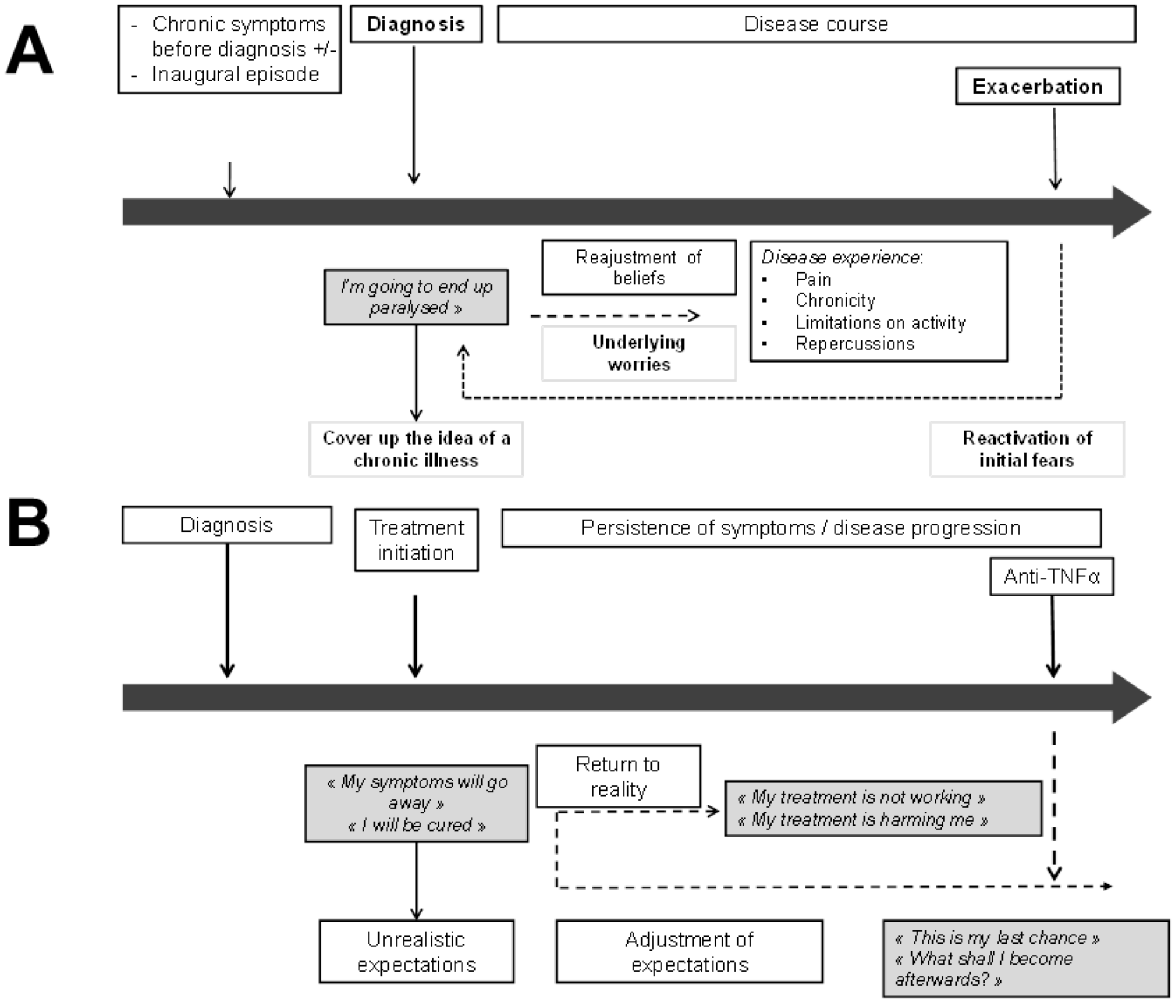
Evolution of beliefs and apprehensions over the disease course. **A:** disease perceptions. **B:** treatment expectations.

It was also observed that the beliefs patients held about their disease varied as the disease progressed (Fig. 2a). An analogous dynamic system of beliefs about treatment expectations was also apparent (Fig. 2b). These findings are consistent with two previous studies performed in England, which showed that patients' perceptions of treatment evolved with the course of disease [15], [16].

Many of the perceptions held by patients about their disease or its treatment could be considered as inappropriate from a medical point of view, such as the belief that diet could influence the risk of exacerbations or that disease-modifying treatments could allow joint structure to be ‘restored’. There is clearly a need for better patient education, and also for perhaps for psychological interventions, to ensure that expectations are realistic and behaviours are appropriate, thereby avoiding distress when expectations are not fulfilled. In order to achieve this, physicians should actively explore the beliefs and apprehensions of their patients in order to dispel inappropriate beliefs and unwarranted worries. In a previous French survey of patients with RA and their physicians, limited concordance between patients' and physicians' perceptions of the impact of their disease on functioning was observed, with physicians underestimating impact for almost half of the patients [22]. With respect to treatments, the physician needs to explain clearly the anticipated benefits and risks of treatment, and to explain the role of non-pharmacological interventions such as physiotherapy. Psychological interventions, such as cognitive behavioural therapy, have also been shown to be useful for improving self-management in RA [23]–[25]. Appropriate treatment expectations result in better treatment adherence and an improved outcome over the long-term. In addition to patient-physician dialogue, other channels for patient education should not be neglected, including patient associations and reliable sources of information on the internet. In the majority of cases, practices to avoid or improve symptoms were started at the patients' own initiative and without any discussion with their physician as to their utility. The patients reported that they felt that it was helpful to be in charge and feel that they were taking active measures to improve their health, and most felt strongly that these activities were beneficial.

In another rheumatological disorder, osteoporosis, inappropriate beliefs about disease are also frequent, particularly in men, notably with regard to susceptibility and to the importance of lifestyle measures [26]. In osteoporosis, inappropriate beliefs are an important barrier to lifestyle behaviours that could potentially improve prognosis, such as increasing dietary calcium intake and physical exercise [26].

An interesting aspect of the study was to evaluate whether there were differences in disease perceptions between patients with RA and those with SpA. In this comparison, it should be borne in mind that the patients with RA were predominantly female, whereas those with SpA were not, so it is possible that potential differences between the two diseases may be contaminated differences between the two groups in terms of gender or other patient or disease features. Overall, perceptions were quite similar between the two groups. Differences were limited to two principal themes. The first related to factors responsible for outbreak of the disease. Patients with RA more frequently proposed an external factor, such as a traumatic or distressing life event or a psychological cause whereas those with SpA tended to focus on an intrinsic vulnerability to disease. This may be related to the fact that the initial clinical manifestations of RA generally have a more abrupt onset than those of SpA, in which symptoms develop insidiously and may take many years to be recognised as SpA [27], [28]. In addition, the association of SpA with HLA variants is more robust than in the case of RA [29]. The second difference related to concerns about progression of disease, with patients with RA being more worried about spread to other joints and those with SpA being concerned about paralysis resulting from joint fusion. Patients with RA were more concerned about future stigma, perhaps because joint deformity is more visible in this disease. In addition, patients with RA more frequently reported recourse to alternative medicine as a way of limiting exacerbations, whereas those with SpA more frequently reported doing sports or exercise.

The study has certain strengths and weaknesses. The strengths include the length of the interview, which allowed multiple themes to be developed in depth and the use of open-ended questions, exclusively in the first phase and predominantly in the second phase, which limits the risk of bias due to potential preconceptions of the investigators and the interviewers. The limitations include the small sample size, potentially leading to inadequate representativeness. However, the fact that we achieved saturation of themes suggest that the sample size is sufficient, and is in any case at the upper end of the range of other qualitative studies in RA [6]. Moreover, the findings have some face validity and reflect concerns that these patients may legitimately have. Although sample representativeness is not as critical an issue in qualitative research as it is in quantitative hypothesis-testing studies, it is hard to assess the representativeness of the patients enrolled in the study. In this respect, it would have been helpful to have more information on the clinical and functional status of enrolled patients, for example in terms of HAQ or BASFI scores or of HLA-B27 status. Unfortunately, such data are not routinely collected in everyday practice in France. The choice of face-to-face interviews may also have introduced a bias if patients found it difficult to bring up sensitive subjects or express negative views in the presence of a third party. Finally, it was not possible in an exploratory survey such as this to collect reliable quantitative data.

The findings of this study open several perspectives for future research. In particular, validated disease-specific patient-reported outcome (PRO) measures would be useful to capture patient's perceptions of disease. In RA, the only disease-specific PRO measures available have been developed to assess quality of life. These include the *Rheumatoid Arthritis Impact of Disease* (RAID) health profile and the twenty-item *Evaluation of Ankylosing Spondylitis Quality of Life* (EASi-QoL) questionnaire [30], [31]. It would, however, be useful to have a PRO that could evaluate patients' fears and beliefs in a quantitative manner, since these could be targeted by specific counselling or educational programmes. In this perspective, the qualitative information collected in the present study, notably that related to fears for the future and perceptions of disease impact, may be useful as a first step to identify the most pertinent themes to be explored when building a patient questionnaire.

Moreover, the findings could help physicians to understand their patients' perceptions of their disease better and thus improve the patient-physician dialogue. This kind of information may make physicians better prepared for identifying and dispelling inappropriate beliefs and behaviours, and for investigating and managing subjective repercussions of disease, such as depression.

In conclusion, patients with RA or SpA hold a core set of beliefs and apprehensions that reflect their level of information about their disease and are not necessarily appropriate. There was often a mismatch between initial perceptions and the reality of the disease, which evolved over the course of the disease. The physician can initiate discussion of these beliefs in order to dispel misconceptions, align treatment expectations, provide reassurance to the patient and readjust disease management. There is clearly a need for improving patient education, which could be addressed through multiple channels, including healthcare professionals, patient associations, dedicated authoritative websites and the media. A clear aspiration for more discussion with the physician was identified in the interviews. Such a dialogue would help improve standards of care in these chronic and incapacitating diseases. In this respect, the development and validation of a tool to document individual patient fears and beliefs would be useful.

## Supporting Information

S1 Table **Examples of questions asked during the interviews in Phases I and II of the survey.**
(DOCX)
