# Correction: Fears and Beliefs in Rheumatoid Arthritis and Spondyloarthritis: A Qualitative Study

**DOI:** 10.1371/journal.pone.0119056

**Published:** 2015-03-05

**Authors:** The PLOS ONE Staff

There is an error in affiliation 3 for author Christophe Hudry. Affiliation 3 should be: AP-HP, Hôpital Cochin, Rheumatology Department, Paris, France.
