# Supplementary material for: Fears and Beliefs in Rheumatoid Arthritis and Spondyloarthritis: A Qualitative Study
Source: PLoS One. 2014 Dec 4;9(12):e114350. doi: 10.1371/journal.pone.0114350 (PMC4256228; doi:10.1371/journal.pone.0114350)
Supplement: S1 Table — Examples of questions asked during the interviews in Phases I and II of the survey. (DOCX) [file pone.0114350.s001.docx]

# SUPPORTING INFORMATION

**Table S1.** Examples of questions asked during the interviews in Phases I and II of the survey.

| *Study Phases I and II* (*open-ended*) |
| --- |
| - Have you personally thought about the possible causes of your own illness, about what could have triggered it or be responsible for it? |
| - If so, what causes have you thought about? |
| - What are your reasons for thinking that this may be important? |
| - Does your illness have any impact on your social life or family life, or on your relationship with friends and family? |
| - If so, could you tell me more about this? |
| - How do you deal with these issues, how do you come to terms with them? |
| - How do you feel about this? |
| - What do you think about the different treatments for your illness? |
| - Has your opinion about your treatment changed? If so, in what way? |
| - Do you have difficulties with taking your medication? If so, what are they? |
| - Could you tell me more about how you cope with these difficulties and how you feel about them? |
| *Study Phase II only* (*semi-directive*) |
| - Some patients tell me that they thought that their eating habits certainly contributed to them getting ill: they thought that eating too much meat, too much milk or unhealthy food was responsible. |
| - Do you think that there is a link between what you eat and your illness? Has this idea ever crossed your mind? |
| - If so, what sort of link? What are your reasons for thinking this? |
| - Are there other things that you think may cause your illness, not necessarily things that your doctor has told you, but ones that you believe in? What sort of things? |
| - A patient with the same illness as you told me that he thought that taking medication for a long time would necessarily have bad effects on him, even though it would treat his disease. |
| - Are there things like this that you believe or worry about with your treatments? |
| - In particular, do you worry about having to take medications for a long time? |
| - If so what do you worry about and why? |
| - Have you talked about these worries to your doctor? Why? |
